# Impurity Controlled near Infrared Surface Plasmonic in AlN

**DOI:** 10.3390/nano12030459

**Published:** 2022-01-28

**Authors:** Quanjiang Li, Jingang Wang, Shenghui Chen, Meishan Wang

**Affiliations:** 1 School of Physics and Optoelectronics Engineering, Ludong University, Yantai 264025, China; lqj@ldu.edu.cn; 2 College of Science, Liaoning Petrochemical University, Fushun 113001, China; jingang_wang@lnpu.edu.cn; 3 School of Integrated Circuits, Ludong University, Yantai 264025, China

**Keywords:** impurity controlled, surface polarirons, plasmon

## Abstract

In this work, we used multi-scale computational simulation methods combined with density functional theory (DFT) and finite element analysis (FEA) in order to study the optical properties of substitutional doped aluminium nitride (AlN). There was strong surface plasmon resonance (SPR) in the near-infrared region of AlN substituted with different alkali metal doping configurations. The strongest electric field strength reached 10^9^ V/m. There were local exciton and charge transfer exciton behaviours in some special doping configurations. These research results not only improve the application of multi-scale computational simulations in quantum surface plasmons, but also promote the application of AlN in the field of surface-enhanced linear and non-linear optical spectroscopy.

## 1. Introduction

Surface plasmons (SPs) are coherent electron collective oscillations (CEO) that propagate along the interface with electromagnetic waves, and are prevalent at the interfaces of different materials [1,2]. SPs are typically observed in nano-scale metal and non-metal interfaces. Due to the nature of the interface and the electromagnetic field mode, some SPs are limited to a small region (LSPRs) [3,4], and some SPs will propagate along various paths (PSPPs) [5,6]. These two different SPs are commonly referred to as localised surface plasmons and propagated surface plasmons, respectively. The former are widely used to enhance weak signals, such as surface-enhanced Raman spectroscopy (SERS) [7,8,9], tip-enhanced Raman spectroscopy (TERS) [10,11,12,13], and surface-enhanced fluorescence. The latter plays an irreplaceable role in remote photocatalyst reactions and integrated optical devices, such as miniature optical waveguides and modulators. The SPs’ materials are widely used in these fields, in which SPs resonance (SPR) is distributed in ultraviolet or visible light regions. For some special applications, such as optical communication, biological detection in vivo, and photon medicine [14,15], SPR in the near-infrared band is more prevalent. However, due to the rapid development of non-linear optics, SPR also plays a very important role in some non-linear optical spectroscopy fields, such as surface-enhanced two-photon excited fluorescence (SE-TPEF) [16,17,18], second harmonic generation (SE-SHG) [19], and coherent anti-Stokes–Raman scattering (SE-CARS) [20]. These applications require that the intensity of femtosecond lasers be enhanced in the near-infrared region, making the development of near-infrared SPs with improved SPR properties imperative. To generate SPs, the real part of a material’s complex dielectric function must be negative in a certain wavelength range, and the imaginary part should be as large as possible. Because almost all metals, especially Ag and Au [21], meet these conditions in this region, these two precious metals are often used in SPs to enhance Raman or fluorescent signals. Therefore, regulating the dielectric function of materials is very important to extend the application of SPs. Heterostructure and doping are often used to regulate a material’s properties. However, obtaining a material’s accurate dielectric function after doping and modification is challenging. Common methods include traditional Drude [22,23,24] models and equivalent media theory [25,26,27]. However, the practical effects of these methods on calculating the dielectric functions of doped and low-dimensional systems are very modest. Because these situations involve quantum mechanical effects, more precise quantum mechanical methods are needed.

Density functional theory (DFT) is an ab initio quantum mechanical algorithm that does not rely on any empirical parameters [28]. It is a reliable condensed matter physics and first-principles theory that can accurately calculate the electronic structure and optical properties of materials [14]. Because exchange-correlation functionals in DFT can thoroughly describe the exchange-correlation effects between electrons in a multi-electron system, this method is universally applicable to materials composed of various elements, and is not limited to the crystal structures and components. The Finite Element Analysis (FEA) is the simulation of any given physical phenomenon using the numerical technique called Finite Element Method (FEM) [29]. AlN is a common, traditional semiconductor material similar to gallium nitride (GaN) [30,31,32]. AlN was first synthesised in 1877. By the 1980s, aluminium nitride was widely used in microelectronics [33,34,35]. The stability of doped nano-cages is evaluated through binding energy calculations [36,37,38,39]. Unlike beryllium oxide, aluminium nitride is non-toxic. Aluminium nitride is treated with metal and can replace alumina and beryllium oxide in many electronic devices. AlN’s energy gap is as high as 6.2 eV, which is measured in vacuum ultraviolet reflectance.

In this work, we conducted theoretical research on the electronic structure, optical properties, and SPR characteristics of a disk array using DFT on alkali doped AlN crystals. After the Li atom replaces the Al atom, the local electric field intensity between the disks at 1300 nm is as high as 10^9^ V/m. This excellent property can be applied in many fields that require near-infrared SPR, such as biomedicine, Raman spectroscopy, non-linear optics, and non-linear surface plasmons. The multi-scale calculation method used in this work, that is, the method of analysing the surface plasmon properties of a doped system through quantum mechanical calculations, can guide theoretical research into the optical properties of materials under the same conditions.

## 2. Materials and Methods

We established an AlN hexagonal lattice using DFT calculations. Two thiophenes are used as the smallest repeating units. The periodic boundary direction is consistent with the polymer’s length direction. A vacuum layer of 15 Å is present in the other two directions. The atomic centre basis set and the GGA-PBE functional [40,41] are calculated using the QuantumATK-2018.06-SP1 software package [42,43]. Full optimisation of the atomic geometry is performed until all of the components of the residual forces are less than 0.05 eV/Å and the total energy converges within 10^−6^ eV. The k-mesh is 7 × 7 × 1 and the cut-off energy is 1200 eV. Using the same cut-off energy in the optical property calculations, LCAO (linear combination of atomic orbitals) is used for the basis set, the k-mesh increases to 15 × 15 × 1, and the self-consistent field convergence limit increases to 10^−8^ eV. With the current calculation experience, the calculation method chosen in this paper is more accurate [29,44,45,46].

## 3. Results and Discussion

### 3.1. Crystal Lattice Structures

**Figure 1:**
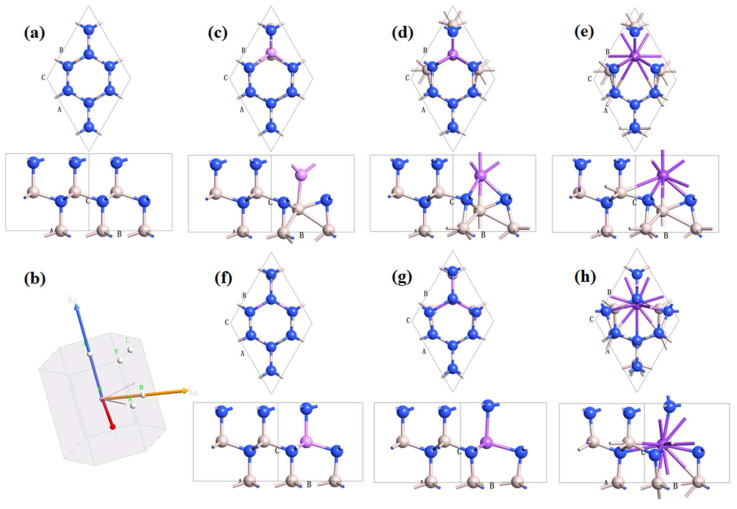
The crystal structure (**a**) and k-space schematic diagram (**b**) of intrinsic AlN. The (**c**–**h**) are the alkali metal substitution (N@Li, N@Na, N@K, Al@Li, Al@Na, Al@K) doping AlN crystal lattices, respectively.

In this work, the geometries and optical properties of different alkali metal atom substitution doping AlN crystal cells are calculated via the ab initio method. The crystal structures optimised by DFT are shown in Figure 1. Alkali metals are doped in two configurations. In the first, alkali metal atoms occupy the position of N atoms in AlN crystals. As shown in Figure 1c–e, one N atom at the same position is replaced with Li, Na, and K atoms, respectively. Since the AlN crystal point group belongs to D3h, it is equivalent to substituting any N atom. After the N atom is replaced by three alkali metals, compared with the intrinsic AlN structure (Figure 1a), the structure after doping undergoes relatively large changes. The alkali metal shifts from the original N atom position and squeezes the Al atom position. Due to the different radii of alkali metal atoms, the coordination mode of the atoms changes considerably. However, when the alkali metal atom replaces the Al atom, the crystal structure inevitably changes, as shown in Figure 1f–h. When Li atoms and Na atoms are substituted with Al atoms, the crystal structure changes only slightly, as shown in Figure 1f,g. This is because the atomic radii of the Li atoms and Na atoms are not significantly different from those of the Al atom. However, the radius of the K atom is relatively large (Figure 1h), so when the K atom replaces the Al atom, the crystal distortion is large.

### 3.2. Electronic Structure

**Figure 2:**
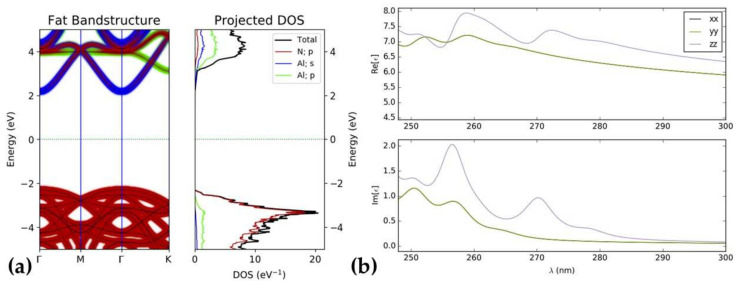
The projected electronic energy band structure, density of states (DOS) spectra (**a**) and frequency dependent isostropic dielectric function (**b**) of intrinsic AlN.

Changes in the crystal structure can cause differences in the electronic structure. As shown in Figure 2a, the electronic band structure of AlN is a classic insulator material. The top of the valence band and the bottom value of the conduction band occur at the gamma point (Γ). The inter-band transition in AlN is a direct transition. The p orbital of the N atomic contribution plays a key role in the valence band, and the conduction band is contributed by the s and p orbitals of the Al atom. The real part of different cartesian component dielectric functions of AlN is greater than zero. Therefore, there are no strong interactions between electromagnetic waves and AlN. There is surface plasmon polarisation on the material surface where the real part of the dielectric function is less than zero and the imaginary part is as large as possible. The imaginary part of the dielectric function is greater than zero, and the real part is greater than zero. The peaks of the imaginary part of the dielectric function of AlN occur in the deep ultraviolet region. These properties limit the application of AlN on SPR, especially the optical and optoelectronic properties.

**Figure 3:**
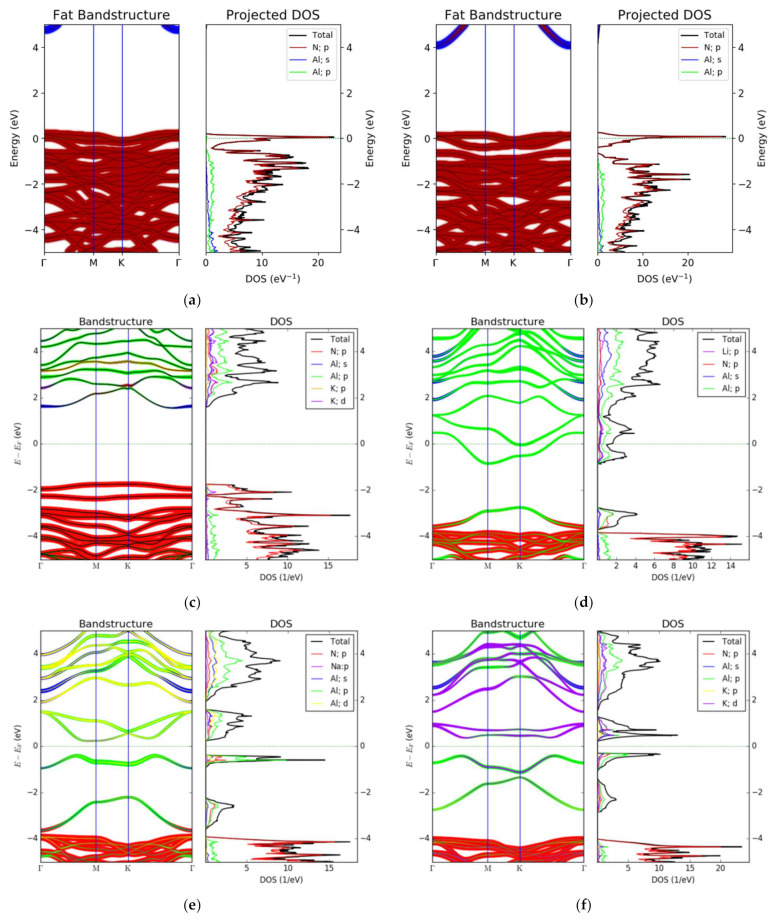
(**a**–**f**) are the electronic structures (projected energy band structure and DOS spectra) of alkali metal substitution (Al@Li, Al@Na, Al@K, N@Li, N@Na, N@K,) doping AlN, respectively.

After doping, the electronic structure of AlN changes dramatically. First, replacing the Al atom position with Li or Na atoms causes the Fermi level to enter the original valence band. Near the Fermi level, no impurity level appears in the forbidden band, as shown in Figure 3a,b. However, the entry of the Fermi level into the valence band significantly affects the electronic transition. According to the DOS spectrum, there is a large state density above and below the Fermi level, so strong intra-band transitions occur. After the K atom replaces the Al atom (Figure 3c), there are no impurity levels in the forbidden band, but some p and d orbital components of the K atom are mixed into the conduction band, and the width of the forbidden band ($E_{g}$) decreases. Although $E_{g}$ decreases, it is still large for electronic transitions (~4 eV). Second, after the Li atom replaces the N atom position, the Fermi level enters the conduction band, as shown in Figure 3d. In addition, there is no small state density near the Fermi level. Therefore, in this configuration, there are no small in-band transitions. Unlike the substitution of Li atoms for Al atoms, this is an in-band transition of the conduction band, and it causes the transition in the p orbit of the Al atom. Third, after the Na and K atoms replace the N atoms, $E_{g}$ significantly decreases. However, the Fermi level remains in the forbidden band, and the density of states near the Fermi level is not small, as shown Figure 3e,f. Thus, there are no small inter-band transitions in these two configurations. Unlike the first five configurations, after the K atom replaces the N atom, the impurity level of the K atom appears in the forbidden band (Figure 3f), and the Fermi energy level is the K atom contribution and the Al atom contribution, respectively. Therefore, not only the inter-band transition but also the charge transfer transition occurs in this doped configuration. The metal–insulator transition depends on the relationship between the Fermi energy level and the energy band. When the conduction band or valence band of the intrinsic material overlaps or interleaves with the Fermi energy level, the metal–insulator transition will occur, and of course topological phenomena may also occur. This is also shown in the dielectric constant below. In summary, when Li atoms and Na atoms are substituted with Al atoms, intra-band transitions on N atoms in the valence band occur. When the Li atoms are substituted by N atoms, there are intra-band transitions on the Al atoms inside the conduction band. When the Na atoms and K atoms are substituted by N atoms, low-band transitions and charge transfers occur in the crystal.

### 3.3. Optical Properties of Doping AlN

**Figure 4:**
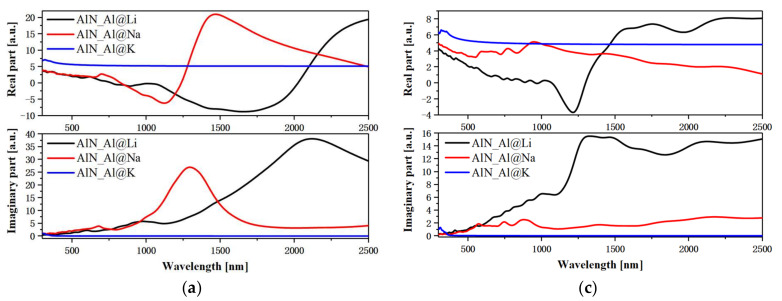

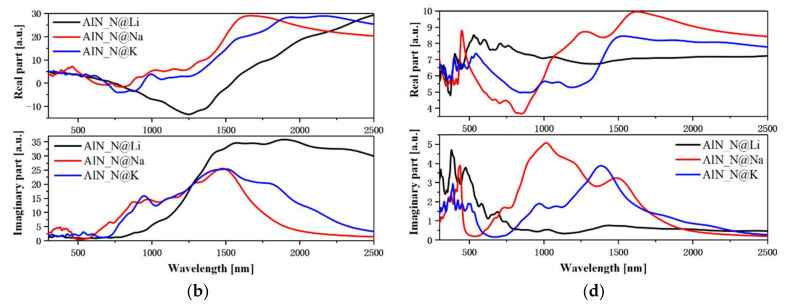
The xx and zz orientation dielectric function of alkali metal substitution (Al@Li, Al@Na, Al@K) (**a**,**c**) and (N@Li, N@Na, N@K,) (**b**,**d**) doping AlN.

Changes in the electronic structure can drastically affect the optical properties. According to the prior description, intra-band transitions and low-energy inter-band transitions occur in many configurations after doping. Therefore, the dielectric functions of the six configurations are calculated separately. Of course, the DFT theory does not consider temperature effects, but the ab initio molecular dynamics theory that considers temperature has little effect on the permittivity. Since AlN is a uniaxial crystal, the dielectric functions in the x and y orientations are equal. The dielectric functions of the three types of AlN and doped AlN are shown in Figure 4, respectively. Figure 4a,c and Figure 4b,d demonstrate cases in which Al atoms and N atoms are replaced, respectively. A necessary condition for the existence of a surface plasmon at a certain wavelength position is that the real part of the dielectric function is less than zero, and the imaginary part is not equal to zero. Because this is the dielectric property of metals, surface plasmons are originally found on rough precious metal surfaces. First, when Li atoms and Na atoms are substituted with Al atoms, the real part of the dielectric function in the xx orientation is less than zero in the near-infrared region, as shown in the top half of Figure 4a. However, when K atoms are substituted with Al atoms, the dielectric function has no properties in the visible and near-infrared regions that can help excite the surface plasmons. This is discussed in Section 3.2. Although the forbidden band width decreases, the inter-band transition remains in the ultraviolet region. For the z orientation shown in Figure 4c, when Li atoms replace Al atoms, the real part of the dielectric function also has a certain negative value, and the imaginary part in the corresponding region is not small. Thus, there may also be surface plasmons in the z direction. The same phenomenon also occurs after three kinds of alkali metal atom substitution types are doped at the N atom position, as shown in Figure 4b. After Li atoms are doped with N atoms, the dielectric function is less than zero in a long near-infrared region, and the value is large, reaching below −10 a.u. However, in N atom and K atom substitution doped N atoms, although there is a certain negative dielectric function at approximately 800 nm, the value is very small. This is because when the two doping configurations are used, the material has the properties of a semiconductor with a relatively small $E_{g}$. Therefore, in this configuration, excitons should appear in the near-infrared region, not surface plasmons.

**Figure 5:**
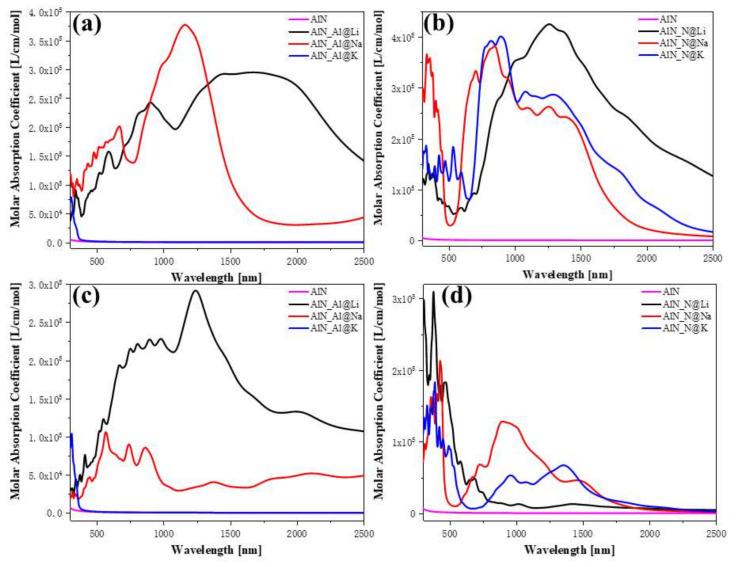
The x and z orientation absorption coefficients of alkali metal substitution (Al@Li, Al@Na, Al@K) (**a**,**c**) and (N@Li, N@Na, N@K,) (**b**,**d**) doping AlN.

The absorption spectrum can characterise the interaction strength between materials and electromagnetic waves. The absorption coefficient is defined as follows:

(1) $$\alpha =\sqrt{\frac{\sqrt{\epsilon_{1}^{2}+\epsilon_{2}^{2}}-\epsilon_{1}}{2}},$$

where $\epsilon_{1}$ and $\epsilon_{2}$ are the real and imaginary parts of the dielectric function, respectively. Therefore, the absorption spectrum can be used to comprehensively analyse the effect of the complex dielectric function. When Li and Na atoms replace Al atoms, they cause strong absorption in the near-infrared region, indicating the presence of surface plasmons, as shown in Figure 5a. When K atoms are substituted, there is almost no absorption coefficient in the visible and near-infrared regions, as demonstrated in Figure 5c. However, when Li atom substitutional doping is performed, since the material also exhibits metallic properties, it also has a strong absorption peak, as shown in Figure 5b,d. After the Na atoms and K atoms replace the N atoms, the system exhibits semiconductor properties, so the absorption spectra are very close.

### 3.4. Plasmonic and Exciton Properties

**Figure 6:**
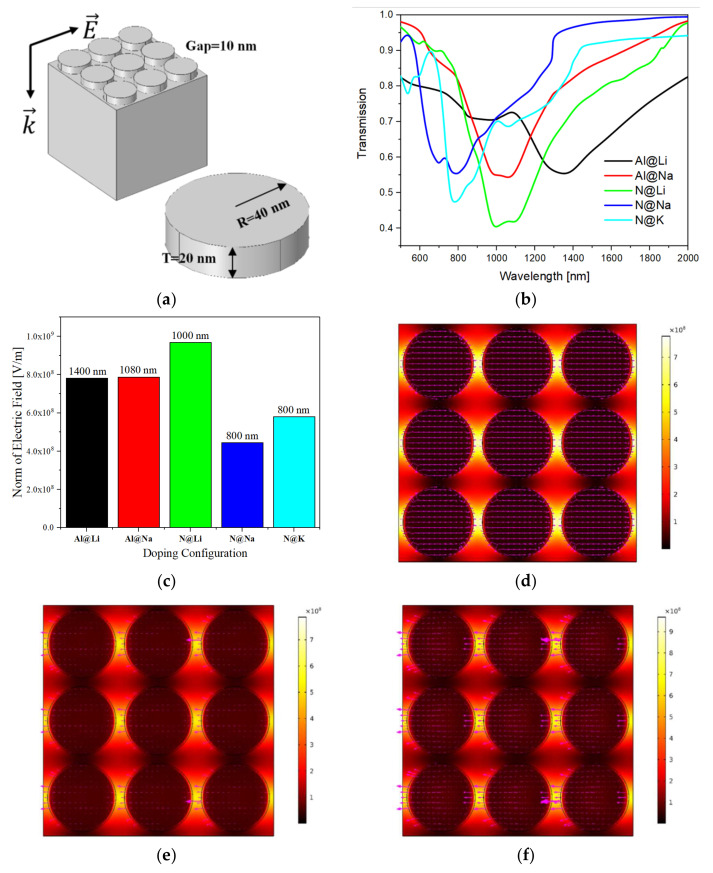

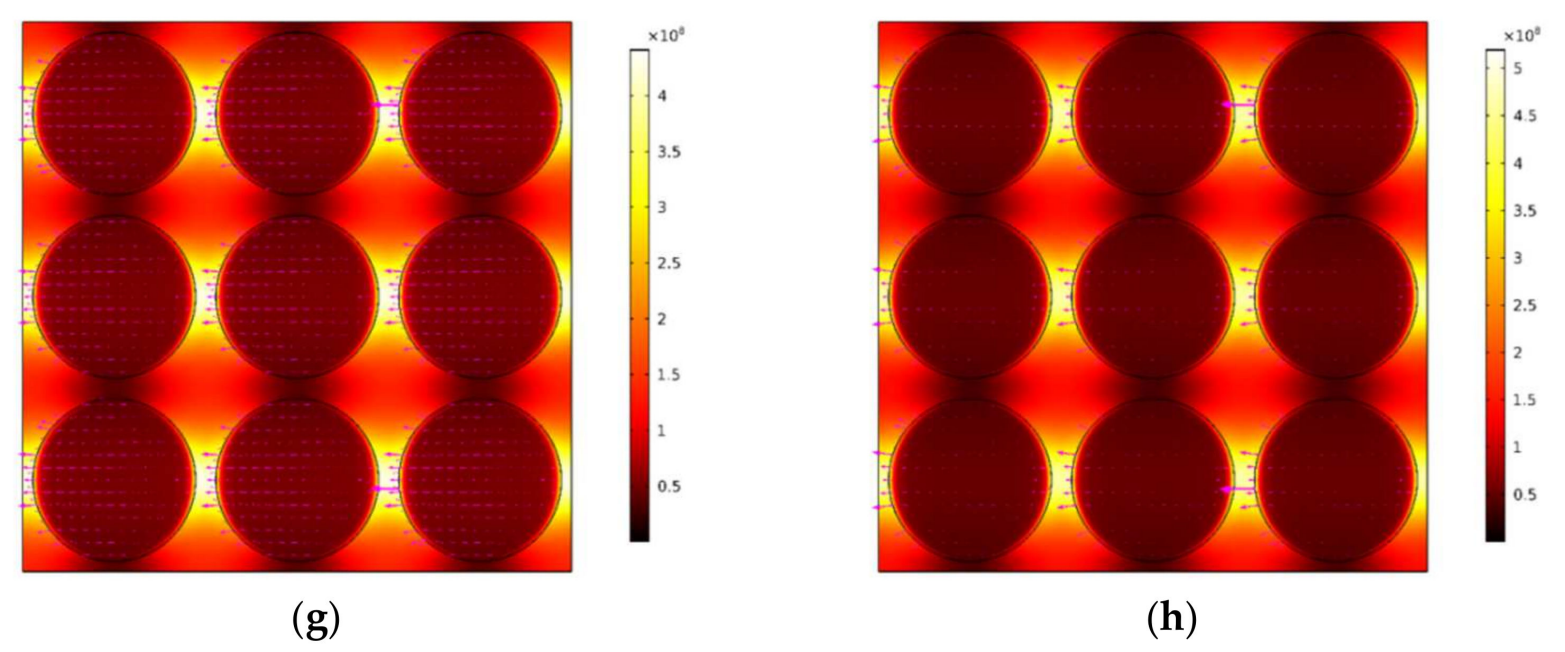
(**a**) The AlN disks array model of FEA analysis. The transmission spectra (**b**) and maximum electric field strength (**c**) of various configurations. (**d**–**h**) The top views of electric field mode of Al@Li, Al@Na, N@Li, N@Na, and N@K doping AlN, respectively. The carmine arrows are the direction of SPs polarization modes.

Based on the prior discussion, a surface plasmon analysis is performed for configurations that may have surface plasmons or exciton behaviour. The AlN disk array model is applied to the analysis of surface plasmons. This is because in materials with surface plasmons, there are hot spots between the disks. Therefore, in the FEA model, a disk array with a radius of 40 nm and a thickness of 20 nm with a gap of 10 nm is placed on the Si surface, as shown in Figure 6a. This is the most common array configuration. Because this work emphasises the effect of the material’s properties on the surface plasmons, the fixed model size only studies the SP properties of different doping configurations. Figure 6b shows the transmission spectra of a disk array with different doping configurations. The transmission spectra peaks indicate the presence of SPR at this position. This is also where the electric field strength is strongest. The figure demonstrates that the positions of the SPR peaks are different, but they all occur in the near-infrared region. Therefore, in addition to the configuration in which the K atom replaces the Al atom, the near-infrared SPR exists in other configurations. The absolute value of the transmission spectrum of the first three configurations in Figure 6b is relatively large because these three configurations have metallic properties. The latter two configurations exhibit semiconductor properties, and the absolute value of the transmission spectrum is relatively small. Correspondingly, the electric field intensity of the first three configurations is strong and the wavelength is longer in Figure 6c, while the electric field intensity of the latter two configurations is weaker and the wavelength is shorter.

According to the prior analysis, there is a charge transfer exciton in the last configuration. As previously mentioned, the nature of the first three configurations and electromagnetic field interaction is SPR, so the electric field model diagram demonstrates that the locality of the electromagnetic field is very strong, as shown in Figure 6d–f. Nevertheless, the nature of the latter two configurations is exciton, so the locality is weaker (the electric field mode in the diagram in Figure 6g,h looks “brighter”). However, although the electric field modes are all similar, the polarisation directions of SPR differ significantly. When Li atoms are substituted with AL atoms, the SPR wavelength is the longest, and the polarisation direction inside the material is very stable, while the net polarisation at the hot spot is almost zero, as shown in Figure 6d. In the latter two SPR materials, the polarisation inside is partially stable, while the polarisation direction at the hot spot is opposite to the polarisation of the incident electromagnetic wave. The polarisation of the two semiconductor-type doped configuration disks is minimal. When Li atoms are doped with N atoms, the real part of the dielectric function has the smallest value, and there is a maximum electric field strength at 1000 nm, which reaches approximately 10^9^ V/m.

## 4. Conclusions

In this work, we conducted multi-scale computational simulation studies on the quantum surface plasmon and exciton properties of AlN doped with alkali metal substitutions at different positions. Depending on the doping type and location, a large SPR intensity exists in the near-infrared region. In Al@Li, Al@Na, and N@Li configurations, SPR occurs at 1400 nm and 1000 nm, respectively. At 800 nm, there are excitons in N@Na and N@K configurations, especially charge transfer excitons in N@K configurations. This paper starts from the atomic scale, simulates the optical properties of AlN doped by quantum mechanical frameworks, and analyses the surface plasmons. AlN modification improves the near-infrared surface plasmon properties (the electric field strength reaches almost 10^9^ V/m), promoting their applications in non-linear optics, CARS, and SRS surface enhancement.

## Data Availability

The data that support the findings of this study are available from the corresponding author upon reasonable request.
